# Galectin-1 Is an Interactive Protein of Selenoprotein M in the Brain

**DOI:** 10.3390/ijms141122233

**Published:** 2013-11-11

**Authors:** Xifeng Qiao, Jing Tian, Ping Chen, Chao Wang, Jiazuan Ni, Qiong Liu

**Affiliations:** 1 College of Life Sciences, Shenzhen Key Laboratory of Microbial Genetic Engineering, Shenzhen University, Shenzhen 518060, China; E-Mails: qiaoxifeng.787@163.com (X.Q.); chenpingszu@126.com (P.C.); 2 Department of Marine Biology, Shenzhen Key Laboratory of Marine Biotechnology and Ecology, Shenzhen University, Shenzhen 518060, China; E-Mails: jing.tianjingtj@gmail.com (J.T.); jzni@szu.edu.cn (J.N.); 3 Changchun Institute of Applied Chemistry, Chinese Academy of Sciences, Changchun 130022, China; E-Mail: raulw2003@163.com; 4 University of Chinese Academy of Sciences, Beijing 100049, China

**Keywords:** selenoprotein M (SelM), galectin-1 (Gal-1), protein-protein interaction, yeast two-hybridization, fluorescence resonance energy transfer (FRET), co-immunoprecipitation, Glutathione *S*-transferase pull-down (GST pull-down)

## Abstract

Selenium, an essential trace element for human health, mainly exerts its biological function through selenoproteins. Selenoprotein M (SelM) is one of the highly expressed selenoproteins in the brain, but its biological effect and molecular mechanism remain unclear. Thus, the interactive protein of SelM was investigated in this paper to guide further study. In order to avoid protein translational stop, the selenocysteine-encoding UGA inside the open reading frame of *SelM* was site-directly changed to the cysteine-encoding UGC to generate the *SelM′* mutant. Meanwhile, its *N* terminal transmembrane signal peptide was also cut off. This truncated SelM′ was used to screen a human fetal brain cDNA library by the yeast two-hybrid system. A new interactive protein of SelM′ was found to be galectin-1 (Gal-1). This protein-protein interaction was further verified by the results of fluorescence resonance energy transfer techniques, glutathione *S*-transferase pull-down and co-immunoprecipitation assays. As Gal-1 plays important roles in preventing neurodegeneration and promoting neuroprotection in the brain, the interaction between SelM′ and Gal-1 displays a new direction for studying the biological function of SelM in the human brain.

## 1. Introduction

Selenium (Se) is an essential trace element for humans and animals. Se deficiency has been found in some regions in the world. Se supplementation is required for people in those areas to prevent various types of diseases, such as man reproduction, cancer, and neurodegenerative diseases [1–6]. The biological effect of selenium in human health is mainly executed via selenoproteins in which Se presents in the form of selenocysteine (Sec), the 21st essential amino acid. This Sec residue is located in the active-site of selenoproteins and encoded by the traditional stop codon UGA in its open reading frame (ORF) of mRNA [7]. Previous studies revealed 25 selenoproteins in human and 24 selenoproteins in mouse [8]. *SelM* was one of those identified via bioinformatics analysis by Gladyshev in 2002 [9,10], and found to be highly expressed in the brain. However, its biological function has not been well studied, especially in the brain.

SelM has 145 amino acids, comprising an endoplasmic reticulum (ER)/Golgi-directing signal peptide in the *N*-terminal, a thioredoxin-like domain in the middle, and an ER retention signal tetrapeptide (H/R/K-X-DL) in the *C*-terminal [10,11]. The Sec residue of SelM is located in the 48th position and present in a CXXU (*i.e.*, Cys-Gly-Gly-Sec) motif upstream of an α-helix. This type of CXXU motif is frequently regarded as a redox center [10]. It has been reported that the activity of antioxidant enzymes in the *SelM*-transgenic mice is higher than the untransgenic mice with Se treatment [12]. The treatment of Se and overexpression of SelM activated the ERK/MAPK pathway, but not the MAPK pathway involving p38 and JNK. It reduced the production of Aβ_1–42_ by down-regulating the activity of β-/γ-secretase and up-regulating the activity of α-secretase [13]. In mouse HT22 hippocampus cells and C8-D1A cerebellum cells, SelM prevented the oxidative damage induced by hydrogen peroxide [14]. *SelM* knock-down in HT22 cells and primary cortical cells caused cell viability to decrease and reactive oxygen species (ROS) to increase [14]. Those reports show that SelM is an antioxidant protein possibly with neuroprotection.

In order to investigate the biological function of SelM in the brain, the interactive protein of SelM was screened by the yeast two-hybrid system and verified by the fluorescence resonance energy transfer (FRET) technique, followed by GST pull-down and coimmunoprecipitation (Co-IP) assays. Galectin-1 (Gal-1) was finally determined to be an interactive protein of SelM.

## 2. Results

### 2.1. Identification of Gal-1 as an Interactive Protein of SelM′

**Figure 1:**
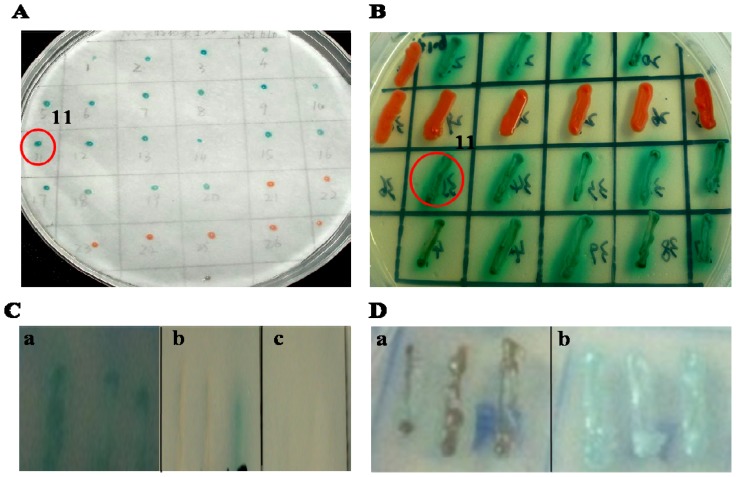
Yeast two-hybrid screening of the human fetal brain cDNA library using Selenoprotein M (*SelM′)* as a bait gene. (**A**) The yeast colonies on the plate SD/-Ade/-Trp/-Leu/-His were picked up and streaked respectively on the squares of filter paper for X-gal display after 8-h incubation. Blue colonies indicated the interaction between prey and bait proteins; (**B**) The yeast colonies were streaked onto the selection plate SD/-Ade/-Trp/-Leu/-His/X-α-gal to check for the interaction between prey and bait proteins (blue colonies); (**C**) Re-transformation experiments. (**a**) the prey plasmid pGACT2-11 and the bait plasmid NpGBKT7-*SelM′* were cotransformed into Y2HGold yeast cells and selected on the plate SD/-Trp/-Leu/-His/-Ade/X-α-gal; (**b**) the prey plasmid pGACT2-11 and empty vector pGBKT7 were cotransformed into the yeast and selected on the plate SD/-Trp/-Leu/-His/-Ade/X-α-gal; (**c**) yeast cells transformed with pGACT2-11 plasmid were restreaked on the plate SD/-Trp/-Leu/-His/-Ade/X-α-gal to check autoactivation of the prey protein; (**D**) Controls for interacting proteins. (**a**) yeasts cotransformed pGBKT7-53 and PGBKT7-Lam as a negative control; (**b**) yeasts cotransformed pGBKT7-53 and pGADT7-T as a positive control (blue color).

Selenoproteins are not expressed in yeast. In order to use the Yeast Two-hybrid System to screen the interacting protein of SelM, human *SelM* gene was site-directly mutated to *SelM′*, where the Sec-coding TGA in *SelM* was changed to Cys-coding TGC in *SelM′*. Cys is an amino acid that has high similarity with Sec in biochemical properties. *SelM′* was then inserted into the vector NpGBKT7 to get the plasmid NpGBKT7-*SelM′*. Before library screening, the plasmid pGBKT7-*SelM′* and the empty plasmid pGADT7 were cotransformed firstly into AH109 yeast cells for the detection of self-activation, using the lithium acetate method. No self-activation and toxicity were observed when SelM′ was expressed in the yeast cells (figures not shown). Then, the human fetal brain cDNA library was screened by *SelM′* via yeast transformation and plate selection. Fourteen clones were grown and displayed blue color on the selection plates SD/-Trp/-Leu/-His/-Ade/X-α-gal (Figure 1B), while 13 clones displayed blue in the filter paper using the X-gal assay (Figure 1A). Among them, 12 clones simultaneously turned blue in both the SD/-Trp/-Leu/-His/-Ade/X-α-gal plate selection and X-gal assay. These 12 DNA fragments originated from the fetal brain cDNA library were respectively amplified, isolated, and retransformed with the bait plasmid NpGBKT7-*SelM′* into Y2HGold yeast cells, followed by the plate selection (SD/-Trp/-Leu/-His/-Ade/X-a-gal). Only one blue colony (marked by a red circle and named 11 in Figures 1A,B) was displayed in the retransformation experiments, whose plasmid from the cDNA library was named pGACT2-11 (Figure 1C(a)). Meanwhile, the yeast cells cotransformed with plasmids pGACT2-11 and pGBKT7 (empty vector) did not display blue (Figure 1C(b)). Yeast cells transformed with plasmid pGACT2-11 were also streaked on the same plates, but they did not display blue, suggesting no autoactivation phenomenon for its protein expression (Figure 1C(c)). Cells co-transfected with plasmids pGBKT7-53 and pGADT7-T were used as positive control for interacting proteins (Figure 1D(b)), while those cotransfected with pGBKT7-53 and pGBKT7-Lam were used as negative control (Figure 1D(a)). The selected positive clones (No. 11) was DNA sequenced and bioinformatically analyzed to be Gal-1.

### 2.2. Verification of the Protein Interaction by FRET Method

In order to investigate whether SelM′ interacts with Gal-1 in living cells, the acceptor photobleaching method was used to measure the fluorescence resonance energy transfer (FRET) between the interactive proteins. The coding sequences of *SelM′* and *Gal-1* genes were inserted into the expression vectors containing the enhanced cyan fluorescence protein (CFP) and yellow fluorescence protein (YFP), respectively. Hela cells were cotransfected by the plasmids pECFP-C1-*SelM′* and pEYFP-C1-*Gal-1*. The cells cotransfected by the vacant plasmids pECFP-C1 and pEYFP-C1 were used as negative controls.

**Figure 2:**
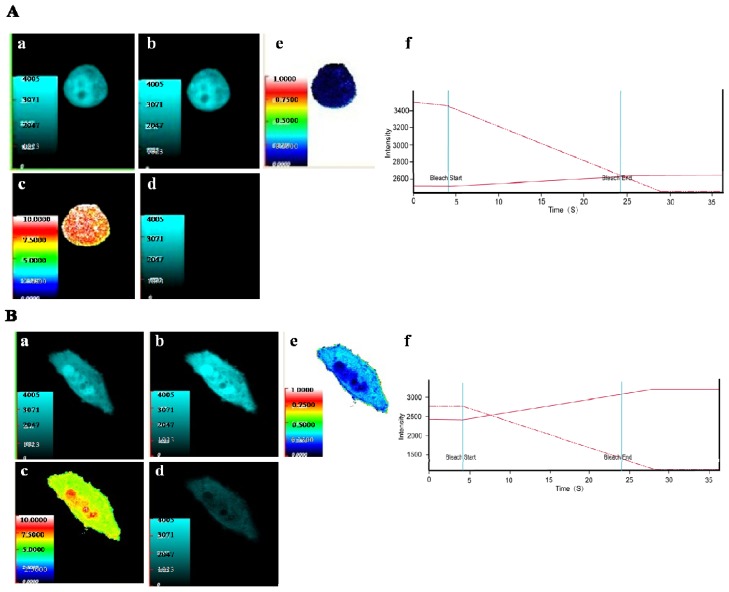
Fluorescence resonance energy transfer (FRET) analysis of the interaction between SelM′ and Gal-1. (**A**) Representative laser scanning microscopy images of HeLa cells cotransfected empty plasmids pECFP-C1 and pEYFP-C1 (negative control). (**a**) The fluorescence image of donor (CFP) before bleaching; (**b**) The fluorescence image of donor after bleaching; (**c**) Diagram of the distance between donor (CFP) and receptor (YFP); (**d**) Donor fluorescence increment before and after bleaching; (**e**) FRET efficiency diagram; (**f**) Photobleaching curve (solid line for donor fluorescence and dashed line for receptor fluorescence); (**B**) Cells co-transfected pECFP-C1-*SelM′* and pEYFP-C1-*Gal-1*. (**a**) The fluorescence image of donor (CFP-SelM′) before bleaching; (**b**) The fluorescence image of donor after bleaching; (**c**) Diagram of the distance between donor (CFP-SelM′) and receptor (YFP-Gal-1); (**d**) Donor fluorescence increment before and after bleaching; (**e**) FRET efficiency diagram; (**f**) Photobleaching curve (solid line for donor fluorescence and dashed line for receptor fluorescence).

Those cells showing both cyan and yellow fluorescence were chosen and imaged through CFP and YFP channels. To perform receptor (YFP or YFP-*Gal-1*) photobleaching, the region of interest (ROI) of cells was bleached at 515 nm for 20 s in YFP channels. The cyan fluorescence of donor (CFP or CFP-*SelM′*) increased significantly after receptor bleaching in the cells expressing CFP-SelM′ and YFP-Gal-1 (Figure 2B(a,b)), but not in the negative control cells expressing CFP and YFP (Figure 2A(a,b)). As shown in Figure 2B(f), the photobleaching curve of cells expressing CFP-SelM′ and YFP-Gal-1 displayed significant increase in donor fluorescence (solid curve) when receptor fluorescence decreased (dashed curve). In contrast, donor fluorescence did not significantly increase in the negative control cells (Figure 2A(f)). The FRET efficiency and the distance between donor and receptor were measured to be 27.3% and 6.35 nm respectively for the cells expressing CFP-SelM′ and YFP-Gal-1, while those for the negative control cells were 3.2% and 8.99 nm respectively. The low FRET efficiency of control cells arose from experimental background. Those FRET results verified the interaction between SelM′ and Gal-1 inside Hela cells.

### 2.3. Verification of the Direct Protein Interaction by GST Pull-Down Assay

**Figure 3:**
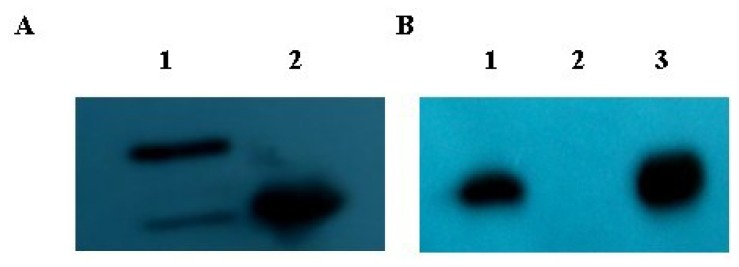
GST pull-down analysis of the interaction between His-tagged SelM′ and GST-Gal-1. (**A**) Western blot (WB) analysis of the expression of GST and GST-Gal-1 with GST antibody. Lane **1**, GST-Gal-1 (42 kDa); lane **2**, GST (27 kDa); (**B**) GST pull-down analysis of the protein interaction with His antibody. Equal amount of GST-Gal-1 (lane **1**) or GST (lane **2**) was bound to glutathione-Sepharose beads. After washing, His-tagged SelM′ bound to GST-Gal-1 was eluted and analyzed by WB with anti-His antibody (lane **1**). Purified SelM′ protein with His tag was used as a positive control (lane **3**).

The open reading frame of *Gal-1* was inserted into the GST expression vector pGEX-5X-1. The fusion protein GST-Gal-1 was expressed in *E. coli* and analyzed by Western blot (WB) as shown in Figure 3A. The PVDF membrane was treated with the enhanced chemiluminescent assay to reveal GST-Gal-1 (lane 1) and GST (lane 2) using GST antibody. As shown in Figure 3B, purified SelM′ with His tag was bound to the GST-Gal-1-binding beads (lane 1) but not the GST-binding beads (lane 2) using anti-His antibody, where purified SelM′ with His tag was used as a positive control to ascertain the reliability of WB analysis (lane 3). Those GST pull-down results verified the direct interaction between Gal-1 and SelM′ *in vitro*.

### 2.4. Verification of the Protein Interaction by Co-Immunoprecipitation Assay

**Figure 4:**
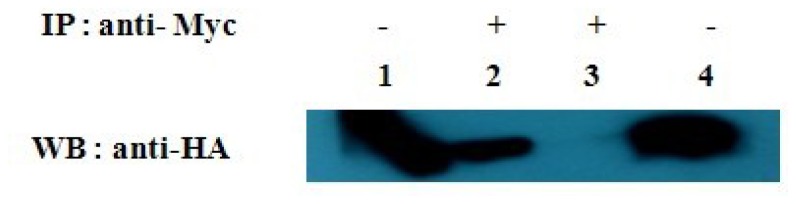
Co-immunoprecipitation analysis of the interaction between Myc-tagged SelM′ and HA-tagged Gal-1. Lanes **1** and **2**, HEK293T cells were cotransfected plasmids pCMV-Myc-*SelM′* and pCMV-HA-*Gal-1*. The cell lysates were analyzed either directly by WB with HA antibody (lane **1**, as a positive control), or by IP with C-Myc antibody and WB with HA antibody (lane **2**). Lanes **3** and **4**, cells were cotransfected plasmids pCMV-Myc and pCMV-HA-*Gal-1*. The cell lysates were analyzed either by immunoprecipitation (IP) with *C*-Myc antibody and WB with HA antibody (lane **3**), or directly by WB with HA antibody as a positive control (lane **4**).

Two plasmids pCMV-HA-*Gal-1* and pCMV-Myc-*SelM′* were constructed and cotransfected into HEK293T cells. Mouse monoclonal c-Myc antibody was used for immunoprecipitation (IP), and monoclonal HA antibody was used for WB. Co-IP results (Figure 4) showed a ~18 kDa protein corresponding to Gal-1 in the cells cotransfected with pCMV-HA-*Gal-1* and pCMV-Myc-*SelM′* (lane 2), but not in the negative control cells co-transfected with pCMV-Myc and pCMV-HA-*Gal-1* (lane 3). Meanwhile, WB analysis was performed for the cells cotransfected with pCMV-HA-*Gal-1* and pCMV-Myc-*SelM′* (lane 1) and the control cells co-transfected with pCMV-Myc and pCMV-HA-*Gal-1* (lane 4). Those results further confirm the interaction between Gal-1 and SelM′ in mammalian cells.

## 3. Discussion

SelM is located inside cells. The *N*-terminal signal peptide within the *SelM* sequence guides SelM to perinuclear structures, including ER and Golgi [10], rather than secreting it into extracellular media. It has been observed that, when the *N*-terminal signal peptide was present, SelM was expressed in ER, whereas no specific localization was seen for SelM in the absence of the signal peptide [10]. In our experiments, the *N*-terminal signal peptide was cut off in order to use the yeast two-hybrid system for screening the human fetal cDNA library. The *N*-terminal-cutoff SelM was also observed to be distributed nonspecifically in the cytoplasm.

It is well known that selenoproteins, including SelM, are difficult to be overexpressed due to the presence of Sec encoded by TGA, a traditional stop codon. Thus, the structure of SelM was analyzed by the mutation of Sec to Cys [15], which did not change the configuration or active-site of SelM. The structure of SelM contains a thioredoxin-like domain and an active-site redox motif arranged in the form of CXXU [15]. The thioredoxin-like domain is composed of a mixed four-stranded β-sheet and three interspersed α-helices. The active-site redox motif of SelM is located between the C terminus of strand β1 and the *N* terminus of helix α1 of the thioredoxin-like doman. Both active-site redox residues (Cys and Sec) are surface accessible. The CXXU motif of SelM is capable of forming a reversible mixed selenenylsulfide bond during the catalytic cycle of oxidation and reduction. Reduction of the active-site selenenylsulfide bond results in localized conformational changes that are centered on the redox motif. Due to the special structure of SelM, the Sec residue in SelM always functions with the adjacent Cys, similar to other protein disulfide isomerases with the redox active site of the CXXC motif. Thus, mutation of Sec to Cys in *SelM* does not change the structure or function of the active-site redox motif, and it should not produce a significant difference in the interaction between SelM and Gal-1.

Gal-1, an endogenous mammalian lectin, is a 14.5-kDa soluble protein present both inside and outside cells, and has both intracellular and extracellular functions [16–19]. The extracellular functions require the carbohydrate-binding properties of dimeric Gal-1 while the intracellular ones are associated with carbohydrate-independent interactions between Gal-1 and other proteins [19]. Human lung fibroblast cells contained high concentrations of intracellular Gal-1, whose characteristics in structure and activity decided its intracellular localization. The thiol groups of Gal-1 in reduced form limited the protein to be located intracellularly [20]. Meanwhile, no signal peptide sequence was found before the *N*-terminal alanine to direct the protein for secretion. The *N*-terminal alanine is blocked with an acetyl group, and the acetylated *N*-terminal amino groups are usually associated with cytoplasmic proteins [19,20]. Interestingly, in most cases the lectin activity of Gal-1 is observed when it is extracellular, while the protein-protein interactions of Gal-1 concern its intracellular functions. The proteins that have been thus far identified which interact in a carbohydrate-independent manner with Gal-1 are not structurally related to each other and do not seem to share any common domains or motifs [19]. The galectin sites that are involved in these interactions have not yet been established. Thus, it is difficult to predict the interacting domains between Gal-1 and SelM, and this work is worth investigating further.

Gal-1 is widely expressed in mouse brain neurons, neural stem cells, and neuroblasts [21,22]. It regulates neural cell fates, such as cell proliferation, differentiation, and death [23–26]. It is essential for generating new neurons and in the recovery from brain damage [17,27]. Gal-1 also plays important roles in adult neural stem cells (NSCs) under both physiological and pathological conditions [28]. Gal-1 deficiency resulted in attenuated proliferation of neural progenitors in the hippocampal dentate gyrus (DG). Astrocytes promote neurogenesis in the adult hippocampus [29] and Gal-1 expressed in activated astrocytes is involved in the promotion of insult-induced neurogenesis in the DG [27]. Gal-1 also induces brain-derived neurotrophic factor (BDNF) in astrocytes to promote adult neurogenesis [30–32]. In addition, Gal-1 is a downstream target upregulated by ΔFosB, a protein induced immediately after ischemia in the DG of hippocampus [23,33], especially in neurons resistant to the injury. It is widely accepted that activated microglia exert dual functions, that is, pro-inflammatory (M1) and anti-inflammatory (M2) [34]. M1 microglia plays a key role in neuronal degeneration [35]. Gal-1 promoted the deactivation of M1 microglia and upregulated the activation of M2 microglia by modulating the signaling pathways of CREB, p38-MAPK, and NF-κB, which eventually prevented neurodegeneration and promoted neuroprotection [35,36]. Gal-1 may thus be considered as a means for the prevention of neuronal loss in cases of injury to the central nervous system [19,37]. SelM is an antioxidative protein that surpresses ROS level and protects neuronal cells [16]. Interestingly, SelM has also been reported to have neuroprotective function in previous reports [14]. In this study, SelM was found to interact with Gal-1, suggesting that the neuroprotection of SelM may be carried out indirectly by Gal-1. A detailed mechanism remains to be investigated in future studies.

## 4. Experimental Section

### 4.1. Materials and Reagents

The yeast strains Y2HGold and AH109, human fetal brain cDNA library and plasmids pGADT7, pGBKT7-53, pGBKT7-Lam, and pGADT7-T were purchased from Clontech (Mountainview, CA, USA). NpGBKT7 was kindly provided by Professor Keke Huo in the Fudan University. The *E. coli* strain TOP10 was stored in our lab. Myc, HA, His, and GST monoclonal antibodies, HRP-goat anti-mouse IgG and Protein-G agarose were purchased from SANTA CRUZ (Santa Cruz, Dallas, TX, USA). DNA-MATE for transfecion was from Bonataike (Shenzhen, China). The pECFP-C1 and pEYFP-C1 vectors were gifted by Professor Shengli Tian in the Shenzhen University.

### 4.2. Gene Amplification and Plasmid Construction

Full-length *SelM* gene was PCR-amplified from the human fetal brain cDNA library. The *N*-terminal of SelM containing 27 amino acids was predicted online to be a transmembrane region, and it was cut off to let SelM possible for yeast two-hybrid screening. Meanwhile, *SelM* was site-directedly mutated to *SelM′* by changing the in-frame Sec-coding TGA to the Cys-coding TGC in the primer, and amplifying the ORF of *SelM* via overlapping PCR. The primers were designed and shown in Table 1. The amplified *SelM′* fragment was used to construct the bait plasmid NpGBKT_7_-*SelM′* for the following yeast two-hybrid assay.

### 4.3. Library Screening via the Yeast Two-Hybrid System

Yeast transformation and two-hybrid screening were performed using the bait plasmid NpGBKT_7_-*SelM′* to screen the human fetal brain cDNA library, following the procedures described in the Yeastmaker™ Yeast Transformation System 2 User Manual and the Matchmaker™ GAL4 Two-Hybrid System 3 & Libraries User Manual (Clontech, Mountain View, CA, USA).

### 4.4. Mammalian Cell Culture

Human embryonic kidney 293T cells (HEK293T) and Hela cells were cultured in Dulbecco’s Modified Eagle’s Medium (DMEM) supplemented with 10% (*v*/*v*) fetal bovine serum and maintained at 37 °C in 5% CO_2_.

### 4.5. Fluorescence Resonance Energy Transfer (FRET) Detection

The FRET method of acceptor photobleaching was performed to validate the interacting proteins. Plasmids pECFP-C1-*SelM′* and pEYFP-C1-*Gal-1* were constructed and sequence-verified. Hela cells were cotransfected pECFP-C1-*SelM′* and pEYFP-C1-*Gal-1* for FRET analysis using laser confocal microscope (OLYMPUS, FV1000, Tokyo, Japan), while plasmids pECFP-C1 and pEYFP-C1 were cotransformed into the cells as the negative control. The method of acceptor photobleaching was performed as described previously [38,39].

### 4.6. GST Pull-Down Assay and Western Blot Analysis

His-tagged SelM′ protein was overexpressed and purified in our lab [40,41]. The ORF of *Gal-1* was cloned from the human fetal brain cDNA library and inserted into the GST expression vector of pGEX-5X-1. Gal-1 protein was expressed in the *E. coli* BL21 (DE3) pLysS cells. Glutathione (GSH)-Sepharose 4B beads were respectively pretreated with the GST or GST-Gal-1 bacterial cell lysate before binding with GST or GST-Gal-1. Approximately 1 mg of GST or GST-Gal-1 was adsorbed to 20 μL of the pretreated beads. His-tagged SelM′ was incubated with the GST/GST-Gal-1 treated beads or non-treated beads (as a control) at 4 °C overnight in the NETN buffer (50 mM Tris-HCl, pH 7.5, 200 mM NaCl, 2 mM EDTA, 0.1% NP-40, with addition of 5 μL 10 mg/mL PMSF before used). After extensive wash with phosphate buffer saline (PBS), the beads were suspended in 2× protein loading buffer, boiled for 5 min, centrifuged at 13,000 rpm, and subjected to SDS-PAGE. Proteins separated on the gel were transferred onto a PVDF membrane. The membrane was immersed in the blocking buffer overnight. The blots were incubated with polyclonal His antibody (1:1000) in TBST (50 mM Tris-HCl, pH 7.4, 150 mM NaCl, 0.1% Tween 20) containing 5% non-fat dried milk for 2 h at room temperature. The membranes were then washed with TBST three times and incubated with HRP-labeled secondary antibodies (1:10,000) for 1 h. Detection of HRP was performed using chemiluminescent substrate from Bonataike (Shenzhen, China).

### 4.7. Co-Immunoprecipitation Assay

HEK293T cells were cotransfected the plasmids pCMV-Myc-*SelM′* and pCMV-HA-*Gal-1*. Forty-eight hours after transfection, the cells were lysed in RIPA solution (Bonataike, Shenzhen, China). The lysates were centrifuged at 13,000 rpm for 30 min at 4 °C. The supernatants were collected and total protein amount was measured using the BCA method. For immunoprecipitation, the supernatant of cell lysate corresponding to 1 mg of total protein was precleared by Protein G-agarose beads to minimize nonspecific binding. Then the precleared supernatnat was incubated with 2 μg of anti-Myc antibody for 1 h at 4 °C, followed by incubation with protein G-agarose beads overnight at 4 °C. The bound proteins were washed thrice with lysis buffer and dissociated with the beads via boiling and centrifugation. The collected proteins were suspended in 2× protein loading buffer, separated by SDS-PAGE and analyzed by Western blot using the primary antibody of anti-HA.

## 5. Conclusions

The interactive protein of SelM in the human brain was investigated in this paper. A human fetal brain cDNA library was screened by *SelM′* using the yeast two-hybrid system. A new interactive protein of SelM′ was screened out and sequence-analyzed to be Gal-1. Then, *Gal-1* ORF was amplified from the fetal brain cDNA library and used for the construction of different plasmids. The interaction between SelM′ and Gal-1 was further verified by the results of FRET, GST pull-down and co-IP. As Gal-1 plays an important role in neuroprotection, the discovery implies that SelM may implement neuroprotection through its interaction with Gal-1. The present paper provides a new direction for studying the biological function of SelM in the human brain.

## Figures and Tables

**Table 1:**
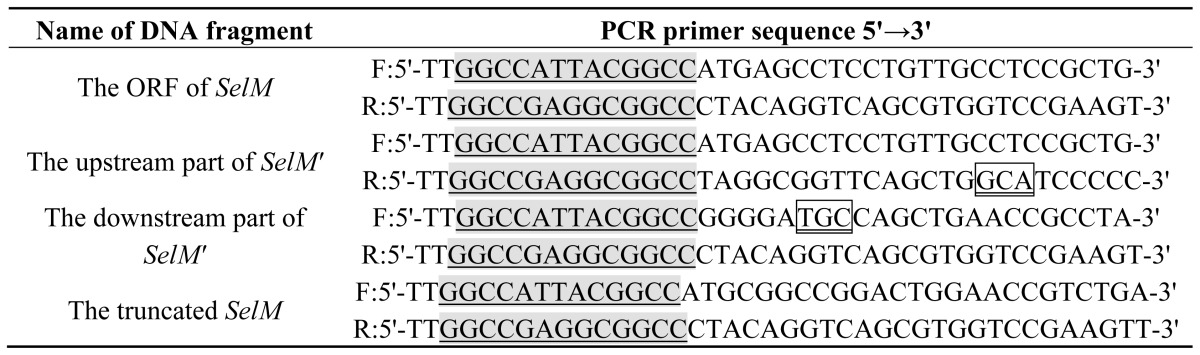
The primer sequences used for PCR amplification of *SelM* and *SelM′*.

F: forward primer; R: reverse primer.
